# Woman; Her Diseases and Remedies

**Published:** 1859-09

**Authors:** 


ART. VII.—*Woman; her Diseases and Remedies. A Series of Letters to his Class.* By CHARLES D. MEIGS, M.D., Professor of Midwifery and the Diseases of Women and Children in the Jefferson Medical College of Philadelphia, etc. Fourth edition, revised and enlarged. 8vo., pp. 706. Blanchard & Lea: Philadelphia. 1859.

THAT this work has been thoroughly appreciated by the profession of this country, as well as of Europe, is fully attested by the fact of its having reached its fourth edition in a period of less than twelve years. Its value has been much enhanced by many important additions, and it contains a fund of useful information, conveyed in an easy and delightful style. Every topic discussed by the author is rendered so plain as to be readily understood by every student; and, for our own part, we consider it not only one of the most readable of books, but one of priceless value to the practitioner engaged in the practice of those diseases peculiar to females.

